# *Treponema pallidum* subsp. *pallidum* TP0136 Protein Is Heterogeneous among Isolates and Binds Cellular and Plasma Fibronectin via its NH_2_-Terminal End

**DOI:** 10.1371/journal.pntd.0003662

**Published:** 2015-03-20

**Authors:** Wujian Ke, Barbara J. Molini, Sheila A. Lukehart, Lorenzo Giacani

**Affiliations:** 1 Department of Medicine, Division of Allergy and Infectious Diseases, University of Washington, Harborview Medical Center, Seattle, Washington, United States of America; 2 Graduate School, Southern Medical University, Guangzhou, PR China; 3 Division of STD, Guangdong Provincial Center for STI & Skin Diseases Control and Prevention, Guangzhou, PR China; 4 Department of Global Health, University of Washington, Seattle, Washington, United States of America; Institut Pasteur, FRANCE

## Abstract

Adherence-mediated colonization plays an important role in pathogenesis of microbial infections, particularly those caused by extracellular pathogens responsible for systemic diseases, such as *Treponema pallidum* subsp. *pallidum* (*T*. *pallidum*), the agent of syphilis. Among *T*. *pallidum* adhesins, TP0136 is known to bind fibronectin (Fn), an important constituent of the host extracellular matrix. To deepen our understanding of the TP0136-Fn interaction dynamics, we used two naturally-occurring sequence variants of the TP0136 protein to investigate which region of the protein is responsible for Fn binding, and whether TP0136 would adhere to human cellular Fn in addition to plasma Fn and super Fn as previously reported. Fn binding assays were performed with recombinant proteins representing the two full-length TP0136 variants and their discrete regions. As a complementary approach, we tested inhibition of *T*. *pallidum* binding to Fn by recombinant full-length TP0136 proteins and fragments, as well as by anti-TP0136 immune sera. Our results show that TP0136 adheres more efficiently to cellular Fn than to plasma Fn, that the TP0136 NH2-terminal conserved region of the protein is primarily responsible for binding to plasma Fn but that binding sites for cellular Fn are also present in the protein’s central and COOH-terminal regions. Additionally, message quantification studies show that *tp0136* is highly transcribed during experimental infection, and that its message level increases in parallel to the host immune pressure on the pathogen, which suggests a possible role for this protein in *T*. *pallidum* persistence. In a time where syphilis incidence is high, our data will help in the quest to identify suitable targets for development of a much needed vaccine against this important disease.

## Author Summary

The study of *Treponema pallidum* subsp. *pallidum* (*T*. *pallidum*) proteins that mediate adhesion to host tissue components is pivotal to understand how the syphilis agent establishes infection and is able to invade virtually every organ system following dissemination from the site of entry. This study focuses on *T*. *pallidum* TP0136, a known plasma fibronectin (Fn) and super Fn binding protein that is heterogeneous in sequence among *T*. *pallidum* isolates. This study shows that TP0136 also mediates attachment to human cellular Fn, that TP0136 conserved NH_2_-terminus is primarily responsible for binding to plasma Fn, but that cellular Fn binding sites appears to be scattered throughout the molecule. Message quantification experiments reveal that *tp0136* transcription is high during experimental syphilis and increases at the time of bacterial immune clearance, suggesting a role for this antigen in counteracting the host defenses during infection, as reported for other Fn binding proteins in other pathogens. Our data deepen the current knowledge of the function of *T*. *pallidum* TP0136 and further support a role for this virulence factor in syphilis pathogenesis.

## Introduction

Syphilis is a sexually transmitted infection caused by the spirochete *Treponema pallidum* subsp. *pallidum* (*T*. *pallidum*). Because syphilis can be rapidly diagnosed with a variety of laboratory tests [1] and efficiently cured by administration of penicillin [2], this disease is often forgotten by our society. Epidemiological data, however, indicate that syphilis is still a relevant global health concern, with an estimated prevalence of 36 million individuals affected worldwide [3], and an incidence of >11 million new cases every year. Although the majority of syphilis cases occur in Latin America, sub-Saharan Africa, and South-East Asia [4], a resurgence of syphilis has been observed over the past decade in the US, Canada, Australia, China, and several European countries [5,6,7,8,9,10]. Furthermore, evidence that congenital transmission of this disease is a leading cause of stillbirths and perinatal deaths in developing areas [11,12], and that patients with syphilis are at increased risk for transmission and acquisition of HIV [13,14], also contributes to make syphilis an important global health problem.

In a naïve host, syphilis infection begins with exposure of mucosal surfaces or epidermal micro-abrasions to *T*. *pallidum* that subsequently begins to replicate at the site of entry. Local bacterial replication and the associated inflammatory and adaptive immune responses induce the appearance of a primary syphilitic lesion (called chancre) teeming with treponemes. The chancre persists until the immune response triggers pathogen clearance by opsono-phagocytosis and allows spontaneous resolution of the lesion [15,16]. Although *T*. *pallidum* cells begin disseminating and invading virtually all organ systems very early during infection [17], local replication is likely favored by the ability of this pathogen to attach to host tissue components. Studies performed in the early 1980’s [18,19] reported *T*. *pallidum*’s ability to adhere to fibronectin (Fn), laminin, collagen IV, and collagen I, suggesting that host extracellular matrix (ECM) is among *T*. *pallidum*’s targets for adhesion. Elucidation of the genome sequence of the Nichols strain of *T*. *pallidum* in the late 1990’s [20] allowed the application of molecular approaches to identify *T*. *pallidum* adhesins, which led to the identification of three Fn binding proteins, TP0136, TP0155, and TP0483 [21,22]. TP0136, in particular, was shown by Brinkman *et al*. [21] to bind human plasma Fn, which is normally found in blood, saliva and other body fluids [23], as well as super Fn, a derivative of plasma Fn that is said to mimic fibrils formed by cellular Fn in the ECM [24]. Whether TP0136 would recognize actual cellular Fn in addition to the plasma variant was not investigated. Similarly, no data exist with regard to the region(s) of the TP0136 protein that mediates binding to Fn, or the relative affinity of TP0136 to Fn types.

Also suggestive of an important role of TP0136 in the pathogenesis of syphilis was the analysis of *T*. *pallidum* transcriptome performed by Šmajs *et al*. [25] on treponemes harvested at peak orchitis from the rabbit host. Results showed that the *tp0136* gene is highly expressed compared to most *T*. *pallidum* genes. However, whether *tp0136* transcript level would differ in treponemes inoculated intradermally (ID) compared to treponemes grown intratesticularly (IT) and whether *tp0136* transcription would vary over time in primary dermal lesions was not explored. Brinkman *et al*. [21] also reported that the putative TP0136 protein sequence is heterogeneous among two syphilis strains (Nichols and SS14), which suggested that TP0136 could be a highly heterogeneous protein. The existence of additional TP0136 variants among *T*. *pallidum* strains was however not explored in previous studies. This work aimed to expand our previous knowledge on *T*. *pallidum* TP0136-Fn interaction dynamics by investigating 1) whether additional TP0136 variants are found among other syphilis strains, 2) whether the *tp0136* transcriptional pattern would vary over time during experimental syphilis infection, 3) how efficiently TP0136 would adhere to cellular Fn compared to plasma Fn, and 4) which region(s) of TP0136 is responsible for binding to Fn. Altogether, the data collected here support the importance of *T*. *pallidum* TP0136 in syphilis pathogenesis.

## Materials and Methods

### Ethics statement

New Zealand White (NZW) rabbits were used for treponemal strain propagation and experimental infections. Animal care was provided according to the Guide for the Care and Use of Laboratory Animals, and procedures were conducted under protocols approved by the University of Washington Institutional Animal Care and Use Committee (IACUC; protocol # 2090–08). No investigations employing human subjects or human samples were undertaken in this study.

### *T*. *pallidum* strain propagation and isolation

Eight *T*. *pallidum* subsp. *pallidum* strains (Nichols Houston, Nichols Seattle, Nichols Dallas, Dal-1, MexicoA, Bal73–1, Seattle81–4, and SS14) were propagated in New Zealand White rabbits by means of intratesticular (IT) inoculation and harvested as previously described [26]. Prior to experimental infection, all rabbits were screened by Venereal Disease Research Laboratory (VDRL) and fluorescent treponemal antibody-absorbed (FTA-ABS) tests to exclude *T*. *paraluiscuniculi* infection. Only VDRL and FTA-ABS negative animals were used. The list of strains employed in this study, along with their isolation dates and sources, is provided in Table 1.

**Table 1:** *T*. *pallidum* subsp. *pallidum* strains used in this study.

Strain Name	Source	Location	Year of Isolation
Nichols Houston^1^	Cerebrospinal fluid	Washington, DC	1912^2^
Nichols Seattle^1^	Cerebrospinal fluid	Washington, DC	1912^2^
Nichols Dallas^1^	Cerebrospinal fluid	Washington, DC	1912^2^
Dal-1^3^	Amniotic fluid	Dallas, TX	1991
MexicoA^4^	Primary chancre	Mexico	1953
Bal73–1^4^	Aqueous humor	Baltimore, MD	1973
Seattle81–4^5^	Primary chancre	Seattle, WA	1980
SS14^6^	Skin	Atlanta, GA	1977

^1^The Nichols Seattle strain was provided by James N. Miller, University of California, Los Angeles, CA. The Nichols Houston strain was provided by Steven J. Norris, University of Texas Health Science Center, Houston, TX. The Nichols Dallas strain was provided by Michael Norgard, University of Texas Southwestern Medical Center, Dallas, TX.

^2^Year refers to the isolation of the parent Nichols strain by HJ Nichols and WH Hough [68].

^3^ The Dal-1 strain [69] was provided by Rob George, CDC, Atlanta, GA

^4^Strains provided by Paul Hardy and Ellen Nell, Johns Hopkins University, Baltimore, MD.

^5^Strain isolated in Seattle by Sheila A. Lukehart, University of Washington, Seattle, WA.

^6^Strain provided by Sandra A. Larsen, Center for Disease Control and Prevention, Atlanta, GA.

### DNA extraction, amplification, and post-amplification procedures

Genomic DNA from the historical strains propagated in our laboratory was obtained as previously described [27]. Briefly, treponemes extracted from infected rabbit testes were separated by slow speed centrifugation from rabbit tissue debris, mixed in a 1:1 ratio with 2X lysis buffer (20 mM Tris, 0.2 M EDTA, 1% SDS) and frozen at-20°C until use or processed using the QIAamp DNA Mini Kit (Qiagen Inc., Valencia, CA) according to the manufacturer’s protocol. Extracted DNA was stored at-20°C until use for PCR and post-amplification applications.

Primers to amplify and sequence *T*. *pallidum tp0136* complete open reading frame (ORF) to perform comparative sequence analysis were designed using Primer3 (http://biotools.umassmed.edu/bioapps/primer3_www.cgi) and are reported in Table 2. Primers were also designed to amplify the complete or partial *tp0136* ORF from the Nichols Seattle and Nichols Houston strains for cloning into the pEXP-5-CT expression vector (Table 2) for producing recombinant proteins. In this case, sense primers annealing to the 5’-terminal region of *tp0136* gene were designed to exclude the sequence encoding the putative signal peptide (aa 1–32, determined using the SignalP4.1 Server, http://www.cbs.dtu.dk/services/SignalP/). For comparative sequence analysis, *tp0136* ORF was amplified in 50 μl final volume using approximately 100 ng of template DNA. Reaction mix contained 200 μM of each dNTP, 1.5 mM MgCl_2_, 0.6 μM of each primer, and 0.5 U of Go-Taq polymerase (Promega, Madison, WI). Cycling conditions were denaturation at 95°C for 10 min, followed by 95°C for 1 min, 60°C for 2 min, and 72°C for 1 min for 45 cycles in total; final extension was at 72°C for 10 min. Successful amplification and size of all products were verified by 1.5% agarose gel electrophoresis. Amplicons from two independent amplifications were sequenced. Prior to sequencing, amplicons were cleaned from residual primers and dNTPs by using the Exo-SAP-IT reagent (Affymetrix, Santa Clara, CA) according to the manufacturer's instruction. Sequencing services were purchased from GeneWiz (GeneWiz Inc., South Plainfield, NJ); sequencing primers are reported in Table 2. Results were analyzed with BioEdit (http://www.mbio.ncsu.edu/bioedit/bioedit.html). Amplification conditions for the *tp0136* complete ORF (w/o the putative signal peptide) and fragments for cloning/expressing were as described above, although the annealing time was reduced to 1 min during the amplification of *tp0136* fragments. Only DNA extracted from the Seattle Nichols and Seattle Houston strains were used in this case. Resulting products were directly cloned into the pEXP-5-CT-TOPO vector according to the provided protocol, and ligation products were used to transform TOP10 competent *E*. *coli* (both vector and cells are from Life Technologies, Carlsbad, CA). Approximately ten insert-containing plasmids were isolated for each of the six constructs using the QIAprep Spin Miniprep Kit (Qiagen) and sequenced to ensure lack of amplification-induced changes before transforming competent *E*. *coli* Rosetta cells (Merck KGaA, Darmstadt, Germany).

**Table 2:** Primers used in this study.

Name	Purpose	Sequence (5’→3’)	Amplicon Size (bp)
**TP0136fl-S**	Amplification of TP0136 from Nichols Houston for protein expression	ATGACGTGCGATTTCACTGG	1389
**TP0136fl-AS1**		CTCGCGGTTCCAGGAGCACG	
**TP0136fl-S**	Amplification of TP0136 from Nichols Seattle for protein expression	ATGACGTGCGATTTCACTGG	1260
**TP0136fl-AS2**		ACTACGTAGATTTTCTGCAC	
**TP0136F1-S**	Amplification of TP0136 Fragment 1 for protein expression	ATGACGTGCGATTTCACTGGC	603
**TP0136F1-AS**		AAACTGTTCGTCCGTGTTTTC	
**TP0136F2-S**	Amplification of TP0136 Fragment 2 for protein expression	ATGGAAAACACGGACGAACAGTTT	468
**TP0136F2-AS**		GGAGTTCGCTTCCAGCTTTAT	
**TP0136F3-S**	Amplification of TP0136 Fragment 3 from Nichols Huston for protein expression	ATAAAGCTGGAAGCGAACTCC	360
**TP0136F3H-AS**		CTCGCGGTTCCAGGAGCACG	
**TP0136F3-S**	Amplification of TP0136 Fragment 3 from Nichols Seattle for protein expression	ATAAAGCTGGAAGCGAACTCC	231
**TP0136F3S-AS**		ACTACGTAGATTTTCTGCAC	
**TP0136-S1**	Amplification of TP0136 for comparative analysis	TCTATTACGAGAAGGAGCGGC	2697
**TP0136-AS1**		GCAGACAAAACCCTCACGATT	
**TP0136-S2**	Sequencing	CCAAGAGGTCAGCGGTTCAA	Not
**TP0136-S3**		GGTTATGAGCATTGCCACCG	applicable
**TP0136-S4**		CTTTTCCACGCCCATCTG	
**TP0136-S5**		GGGGCTGTGGAAGTTTGACA	
**TP0136-AS2**		TGTCAAACTTCCACAGCCCC	
**TP0136-AS3**		CAGAATGGGCGTGGAAAAG	
**TP0136-AS4**		CGGTGGCAATGCTCATAACC	
**TP01365-AS5**		TTGAACCGCTGACCTCTTGG	
**TP0574qPCR-S**	Quantification of TP0574 message	CGTGTGGTATCAACTATGG	313
**TP0574qPCR-AS**		TCAACCGTGTACTCAGTGC	
**TP0136qPCR-S**	Quantification of TP0136 message	GTCGGAAGTGCCCATTAAAA	190
**TP0136qPCR-AS**		GGTGGCAATGCTCATAACCT	

### Recombinant protein expression

**Fig 1:**
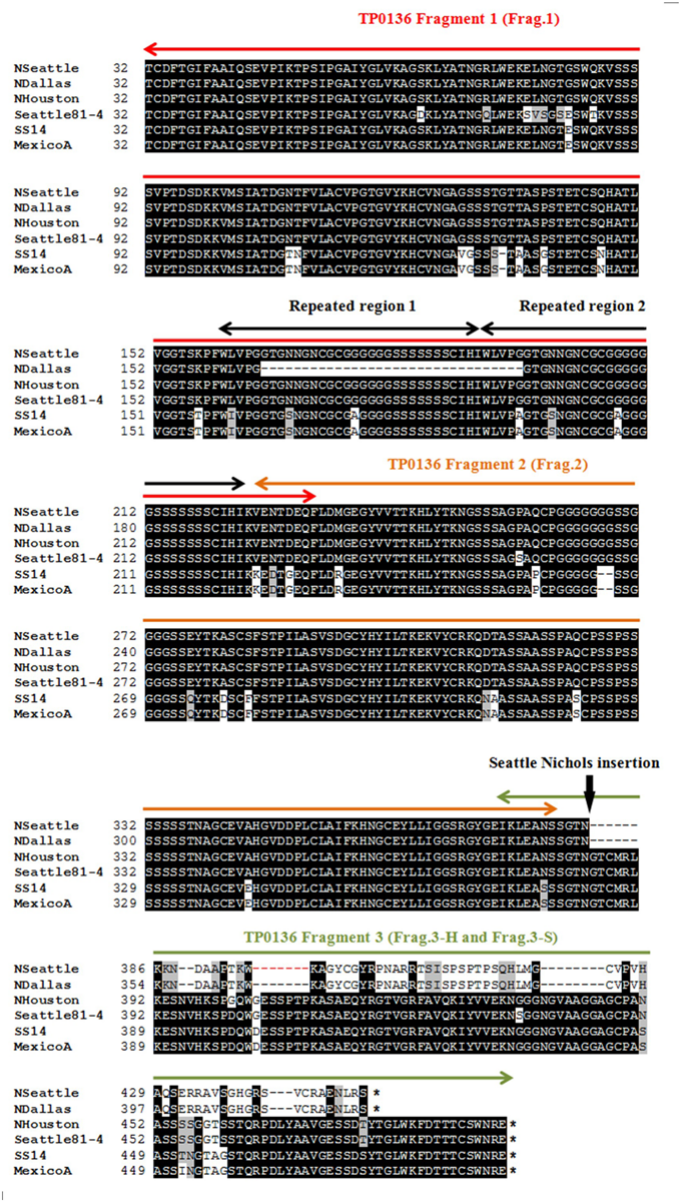
Comparative analysis of predicted TP0136 proteins. Predicted amino acid sequences of TP0136 (without aa 1–31, belonging to the signal peptide) from eight *T*. *pallidum* strains (Nichols Houston, Nichols Seattle, Nichols Dallas, Dal-1, MexicoA, Bal73–1, Seattle81–4, and SS14). Only one sequence representative of each identified TP0136 variant is represented here. Black shading indicates 100% sequence identity, gray shading reflects functionally similar amino acids, and no shading is indicative of sequence similarity below 50%. Dal-1 sequence is identical to Nichols Seattle, while Bal73–1 sequence is identical to Nichols Houston. Nichols Dallas is identical to Nichols Seattle with the exception of a 32 amino acid deletion. Seattle81–4 sequence is unique to this strain. Red horizontal arrows delimit TP0136 Fragment 1 (201 amino acids, with a 7 amino acid-overlap with Fragment 2). Orange horizontal arrows indicate Fragment 2 (156 amino acid, with a 7 amino acid-overlap with both Fragment 2 and Fragment 3, respectively). Green horizontal arrows: Fragment 3 (77 and 120 amino acids from Nichols Seattle and Nichols Houston, respectively). Black horizontal arrows indicate a repeated region (partially deleted in Nichols Dallas). The vertical black arrow indicates the location of the insertion in the Nichols Seattle TP0136 gene (also in Nichols Dallas and Dal-1genes) that causes a shift in the reading frame of the gene and early termination. The nucleotide sequence of the insertion was previously shown to contain donor sites for the variable gene *tprK* [37]. Asterisks (*) indicate position of the stop codons.

A total of six recombinant proteins were expressed for our studies. A schematic is provided in Fig. 1. These included full length TP0136 from Nichols Houston (TP0136-H) and from Nichols Seattle (TP0136-S), TP0136 Fragment 1 and 2 (Frag.1/Frag.2, identical in both the Houston and Seattle variants, and corresponding to the protein NH_2_-terminal and central regions, respectively), TP0136 Fragment 3 from Nichols Houston (Frag.3-H), and Nichols Seattle (Frag.3-S), corresponding to the diverse COOH-termini of these proteins. Protein expression was performed at 25°C for 3 days in ZYM auto-inducting media [28] supplemented with 100 μg/ml of ampicillin for TP0136-H and TP0136-S. TP0136 Frag.1, Frag.2, Frag.3-H, and Frag.3-S were expressed in LB supplemented with 0.1 mM IPTG and 100 μg/ml of ampicillin at 25°C for 24 h. Protein expression and correct size of products were confirmed by immunoblotting with anti-6XHis antibodies (Sigma, St. Louis, MO) after protein separation by SDS-PAGE and transfer to nitrocellulose as described [29]. All expression procedures yielded insoluble proteins, as assessed by SDS-PAGE/immunoblotting using pellets from sonicated *E*. *coli* cells. For recombinant protein purification, *E*. *coli* cells were pelleted by centrifugation, sonicated using the GEX 130 Ultrasonic Processor (Cole-Parmer, Vernon Hill, IL) and pelleted again. Supernates were discarded and the insoluble fractions resuspended in 1X binding buffer (0.5 M NaCl, 20 mM Tris-HCl, 5 mM imidazole, pH 7.9) containing 6M guanidine-HCl (for both TP0136 full length variants) or 6M urea (for all TP0136 fragments) and incubated overnight at 4°C on a magnetic stirrer. After centrifugation at 20,000 rpm in a Sorvall RC-5B Refrigerated Superspeed Centrifuge (Thermo Scientific, Rockford, IL), pellets were discarded and supernates filtered through a 45 μm filter. Resulting samples were loaded onto affinity chromatography columns (Bio-Rad, Hercules, CA) loaded with 7 ml of Ni-NTA agarose (Qiagen). Wash step was performed with a total of 10 ml of 1X wash buffer (0.5 M NaCl, 20 mM Tris-HCl, 20 mM imidazole, pH 7.9, with 6M guanidine-HCl or Urea) and eluted using 10 ml of 1X elute buffer (0.5 M NaCl, 20 mM Tris-HCl, 500 mM imidazole, pH 7.9 with 6M guanidine-HCl or Urea). Eluate was collected in 1 ml-fractions. Elute fractions were analyzed by SDS-PAGE and staining with Simply-Blue Safe Stain (Life Technologies) to determine the fractions devoid of contaminating proteins. To undergo SDS-PAGE, an aliquot from fractions containing guanidine-HCl was treated with trichloroacetic acid (TCA)/acetone to precipitate proteins. Fractions deemed the purest by SDS-PAGE were mixed and dialyzed at 4°C in dialysis cassettes (Thermo Scientific, Rockford, IL) with the appropriate molecular weight cut-off against 1X PBS containing gradually decreasing concentrations of urea or guanidine-HCl (from 3M to none). Self-refolding of recombinant TP0136 during dialysis was reported by Brinkman *et al*. [21]. Following dialysis, refolded soluble proteins were separated from the precipitated insoluble fraction by centrifugation and their concentrations evaluated using a bicinchoninic acid (BCA) assay kit (Thermo Scientific) according to the manufacturer's instruction. Recombinant proteins were stored at-80°C until use.

Recombinant *T*. *pallidum* σSigma^70^ (σ^70^, encoded by the TP0493 gene) was expressed as a soluble recombinant protein in *E*. *coli* using the same protocol for expression and purification previously used for another *T*. *pallidum* σ factor (σ^24^, encoded by the TP0092 gene) [27]. TP0493 gene was amplified from Nichols DNA. Resulting amplicon was directly cloned into the pEXP5-CT/TOPO expression vector (Life Technologies) according to the manufacturer’s instructions. A suitable plasmid was selected after assessment of correct orientation and accuracy of the insert sequence. The pEXP5-CT/TOPO vector allows for expression of a recombinant protein without any accessory fusion sequence, with the exception of a carboxyl terminal 6xHis tag preceded by two amino acid residues (Lys-Gly).

### TP0136 transcript quantification

Skin lesion biopsies for quantification of *tp0136* message were obtained from three rabbits experimentally infected with the Nichols Seattle strain. Rabbits were inoculated ID at ten sites on their clipped backs with 10^6^ cells/site. Fur was clipped daily and lesion appearance and dimensions were recorded daily. A biopsy from the leading edge of a lesion was obtained from each rabbit every three days for 30 days. Biopsies were minced with a scalpel and further homogenized using a pestle in phenol-based Ultraspec RNA Isolation Reagent (Biotecx Laboratories, Inc., Houston, TX). Samples were stored at-80°C or processed for total RNA extraction and DNaseI treatment to obtain DNA-free RNA as described [30]. Reverse transcription was performed with the Superscript III First-Strand Synthesis System using random primers as previously reported [30]. Message quantification was performed according to real-time qPCR protocols already established in the laboratory using a relative quantification approach with external standards and normalizing the mRNA level of the treponemal target gene to the mRNA level of TP0574 gene (encoding the 47 kDa lipoprotein) in the same sample [30]. Primers for amplification of a 190-bp conserved fragment of *tp0136* for quantification and construction of a standard were designed using Primer3 and are reported in Table 2. For making the standard, the *tp0136* amplicon was cloned into the pCR2.1 vector (Life Technologies) according to instructions and used to transform TOP10 cells. Insert-containing plasmids were sequenced to ensure lack of amplification-induced nucleotide changes, and purified plasmid was linearized by restriction digestion using HindIII (New England Bioloabs, Ipswich, MA). Linearized plasmid was purified using the QIAquick PCR Purification Kit (Qiagen), and DNA concentration was estimated with a NanoDrop spectrophotometer (NanoDrop Technologies Inc., Wilmington, DE). Concentration in copies/μl was calculated according to the size of the plasmid plus insert. To create the standard, linearized plasmid was serially diluted to span a concentration range of 10^0^–10^6^ copies/μl. TP0574 primers and plasmid standard have been previously described [30]. Real-time qPCR was performed in a LightCycler 2.1 instrument (Roche, Basel, Switzerland) using the Fast Start DNA Master^*Plus*^ SybrGreen Kit (Roche) according to the manufacturer’s instructions. Each sample was amplified in triplicate. Amplification data were analyzed with the LightCycler software version 3.5 (Roche) to extrapolate the copy number of the target from the reaction crossing points. The data were reported as the mean values ± standard error (SE) for triplicate experiments. Differences between levels of *tp0136* message (normalized to *tp0547*) at different time points during infection were compared using ANOVA and Newman-Keuls Multiple Comparison Test with significance set at *p*≤ 0.05.

### Fibronectin binding assays

All TP0136 recombinant proteins (two full length variants and four fragments) were tested in Fn binding assays as described by Brinkman *et al*. [21]. In our study, however, adhesion to human cellular Fn was tested in addition to plasma Fn. Additionally, we evaluated the relative affinity of TP0136 for Fn binding using concentration-dependent assays, and investigated the location(s) of the Fn binding site(s) on TP0136. Briefly, 96-well ELISA plates (Nunc, Rochester, NY) were coated with 1.5 μg/well of either human plasma or cellular Fn (both from Sigma). After blocking all wells with 3% BSA, increasing concentrations (ranging from 1 to 10 μM) of purified recombinant TP0136-H, TP0136-S, Frag.1, Frag.2, Frag.3-H, and Frag.3-S and the four TP0136 recombinant fragments in PBS were added to the Fn-coated wells and incubated for 16 h at RT. *T*. *pallidum* recombinant σ^70^ protein was used as negative control for binding to Fn. Following incubation with recombinant proteins, wells were washed four times with PBS-0.05% Tween-20 (PBST), and 100 μl of mouse monoclonal anti-6xHis antibody (Sigma) diluted 1:1,000 in PBS were added to each well. Following incubation for 16 h at RT, wells were washed again; 100 μl of alkaline phosphatase-conjugated goat anti-mouse IgG (Sigma) diluted 1:2,000 in PBS were added to each well and incubated for 6 h at RT. p-Nitrophenyl phosphatedisodiumhexahydrate (pNPP, Sigma) was used as a colorimetric substrate for detection of recombinant protein binding to Fn. Absorbance was read at 405 nm with a microplate spectrophotometer MAX 250 (Molecular Devices, Sunnyvale, CA). Each experiment was repeated three times to ensure reproducibility of results. Each time, each sample was tested in triplicate. The data were reported as the mean absorbance values at 405 nm ± SE for triplicate experiments. Binding to Fn of the recombinant proteins at different concentrations was compared to equal concentrations of the negative control (σ^70^) by ANOVA with significance set at *p*≤0.05.

### Inhibition of *T*. *pallidum* binding to fibronectin

The capacity of recombinant TP0136 proteins to inhibit adhesion of viable *T*. *pallidum* to cellular and plasma Fn was tested similarly to the method of Cameron *et al*. for other *T*. *pallidum* putative Fn-binding proteins [22,31]. For this assay, Lab-Tek II chamber glass slides (Nunc) were coated with 4 μg of either human plasma Fn or cellular Fn (Sigma) per well and blocked with 3% BSA. Slides were then incubated with either 200 or 800 pmol of each recombinant TP0136 protein, *T*. *pallidum* recombinant σ^70^, or PBS. After washing with saline, 4.5×10^7^ freshly harvested treponemes were added to each well and slides were incubated for 2 h at 34°C under microaerophilic conditions. Oxygen was removed by flushing the incubation chamber with a mixture of compressed N_2_ (95%) and CO_2_ (5%), as described by Fieldsteel *et al*. [32]. Wells were then gently washed 10 times with saline to remove unattached treponemes. Well separators were removed and attached treponemes were counted by darkfield microscopy in 10 fields per well. The observer was blinded to the conditions in each well. Data are reported as the mean number of *T*. *pallidum* cells bound in 10 fields ± standard error of the mean (SEM). Experiments were repeated twice using each time triplicate wells per condition. Significance was assessed by comparing wells coated with recombinant proteins to the PBS control wells with Student’s unpaired two-tailed t-test and significance set at *p*≤0.05. For comparison between different protein concentrations ANOVA test was used.

### Production of anti-TP0136 immune sera and antibody inhibition assay

Antibodies to recombinant TP0136-H and TP0136-S were produced by immunizing six adult NZW rabbits (three per antigen) with a total of 100 μg of purified recombinant proteins per immunization, suspended in TiterMax Gold Adjuvant (Sigma) according to the manufacturer’s instructions. After the initial injection, 2 booster immunizations were given at 3-week intervals. Immune sera were collected immediately before, and 24 days after, the last booster immunization. Heat-treated (56°C for 30 min) sera from replicate immunized animals, sera collected from the same rabbits prior to immunization (normal rabbit serum, NRS), and *T*. *pallidum*-infected rabbit serum (IRS) were combined into respective pools and adsorbed with a BL21 *E*. *coli* lysate in 5% NFM/PBS at 4°C for 16 h. Adsorbed antisera were collected following centrifugation at 10,000 rpm in a microcentrifuge for 10 min. Reactivity of serum pools was tested by ELISA against recombinant antigens. For this procedure, purified recombinant proteins were diluted to 10 nM and 50 μl were used to coat wells of ELISA plates. Wells were blocked with 3% nonfat milk (NFM) for 2 h at RT. One-hundred microliters of adsorbed test and control sera were added to wells of the coated plates, and incubated for 16 h at 4°C. The plates were then washed three times with PBST before addition of 100 μl of a 1:2000 dilution of a goat α-rabbit IgG conjugated with alkaline phosphatase (Sigma) in 1% NFM-PBS for 1.5 h at RT. Colorimetric detection was performed with pNPP as described above. Each experiment was repeated three times to ensure reproducibility. Each time, each sample was tested in triplicate. Bars represent the absorbance at 405 nm ± SEM for triplicate samples. Significance between reactivity to single peptides was assessed by Student’s unpaired two-tailed t test with significance set at * *p<*0.05.

Antibody-mediated inhibition of treponemal adherence to human plasma and cellular Fn was tested as described by Cameron *et al*. [31], with anti TP0136-H or anti TP0136-S specific antiserum. For these assays, Lab-Tek II chamber glass slides (Nunc) were coated with 0.75 μg/well of plasma or cellular Fn and blocked with 3% BSA. Following well washing with PBS to remove excess BSA, 4.5×10^7^ treponemes that had been previously incubated for 2 h at 34°C under microaerophilic conditions as mentioned above with anti-recombinant serum pools at a final serum concentration of 20% were added to each well. NRS and IRS were used as controls. All sera were previously adsorbed against *E*. *coli* lysates as described above. Treponemes were permitted to bind to Fn for 2 h at 34°C under anaerobic conditions. After 10 washes with saline, attached spirochetes were visualized and counted by dark-field microscopy as described above. Data are reported as the mean number of *T*. *pallidum* cells bound to 10 fields ± SEM. Experiments were repeated twice using each time triplicate wells per condition. Inhibition of binding by antisera was compared with the Student’s unpaired two-tailed t-test with significance set at *p*≤0.05.

## Results

### Comparative analysis of *tp0136* sequences

Previous work by Brinkman *et al*. [21] showed 92% and 94% sequence identity and similarity, respectively, between the Nichols and SS14 strains of *T*. *pallidum*. Although these calculations were based on genome sequences recently found to carry a significant number of errors [33], they still hold true when sequences from the corrected genomes are compared. Our investigation of the genetic diversity of *tp0136* was extended to seven additional *T*. *pallidum* strains (Nichols Houston, Nichols Seattle, Nichols Dallas, Dal-1, MexicoA, Bal73–1, Seattle81–4, and SS14). For all these strains, including those whose genome is available [33,34,35,36], *tp0136* was re-sequenced in the laboratory. As a result, four new *tp0136* variants in addition to the two already reported were found, which confirms *tp0136* inter-strain variability. The predicted amino acid sequences of all TP0136 variants identified (without aa 1–31, belonging to the protein signal peptide) are depicted in Fig. 1. Results showed identical TP0136 sequences for Nichols Seattle and Dal-1. Nichols Dallas sequence was identical to Nichols Seattle, with the exception of the deletion of a 32 amino acid repeated sequence in the Nichols Dallas protein. Diversity between the Nichols Seattle/Dallas and Nichols Houston sequences is due to a 198 bp insertion in the Nichols Seattle/Dallas gene sequences that also causes a frame shift and premature protein termination. This insertion was earlier shown by our group to contain donor sequences for the *tprK* gene of *T*. *pallidum*, known to undergo gene conversion mediated by recombination of donor sequences into the gene expression site [37]. The difference in the predicted TP0136 protein sequence between Nichols Seattle and Nichols Houston prompted us to examine its effect on fibronectin binding capacity of these two variants (see below). Nichols Houston and Bal73–1 were also found to carry identical TP0136 sequences. With the exception of one amino acid substitution, MexicoA and SS14 strain sequences were found to be identical to each other. The Seattle81–4 TP0136 was unique compared to the other isolates.

### TP0136 is expressed throughout experimental infection in the rabbit model following intradermal inoculation of *T*. *pallidum*

**Fig 2:**
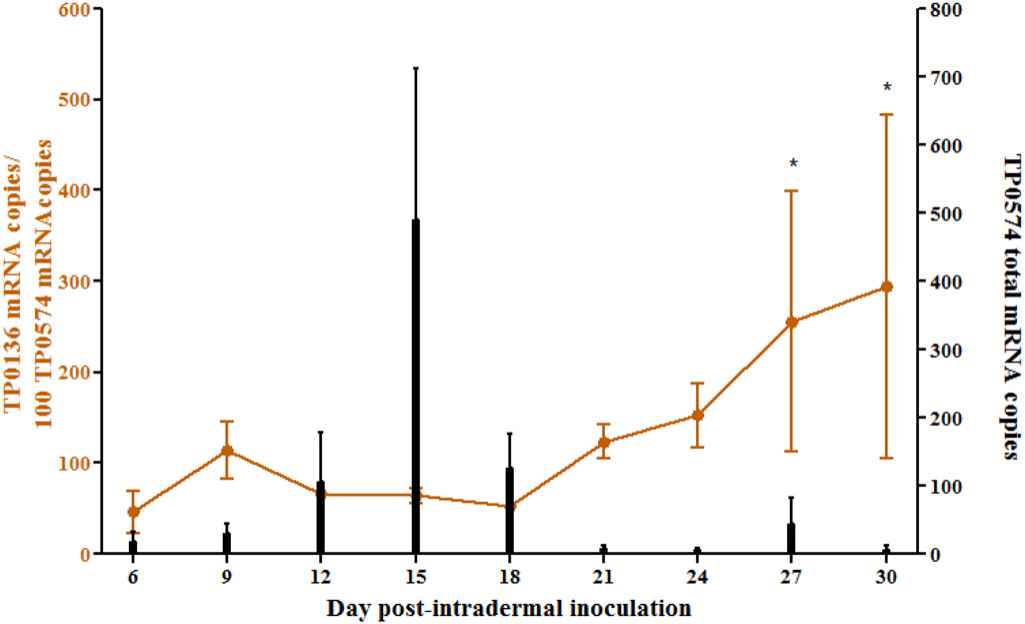
TP0136 message quantification during experimental infection. For message quantification, a biopsy from the leading edge of a dermal lesion was obtained from each of the three infected rabbits every three days for 30 days. Each sample was amplified in triplicate. The data were reported as the mean values ± standard error (SE) for triplicate experiments. Left *y* axis shows real-time qPCR analysis of TP0136 message normalized to TP0547 mRNA (orange line) during progression of primary syphilitic lesions in the rabbit model. Although biopsies were obtained at day 0 and day 3 as well, no message quantification was possible from these samples. Newman-Keuls Multiple Comparison Test was used to assess significant differences in TP0136 message level between time points (*p<0.05) whenever a significant difference between sample means was found by ANOVA. Right *y* axis shows absolute quantification data for TP0574 message (black bars), reflecting absolute *T*. *pallidum* burden.

Quantitative analysis of *tp0136* mRNA expression in primary dermal lesions of rabbits infected with the Nichols Seattle strain showed that transcription of *tp0136* is approximately at the same level as *tp0574* during early lesion development (day 6–18 post-infection), then subsequently increases (day 21–30 post-infection) (Fig. 2). *tp0136* message levels calculated at day 27 and 30 were significantly higher with respect to earlier time points, but not with respect to each other. *tp0574* absolute message level (black bars; Fig. 2) reflects the increase and decline of treponemal burden in experimental lesions due to initial growth of the organism followed by reduction in treponemal numbers due to the host’s adaptive immune response to *T*. *pallidum*.

### TP0136 binds both plasma and cellular fibronectin

**Fig 3:**
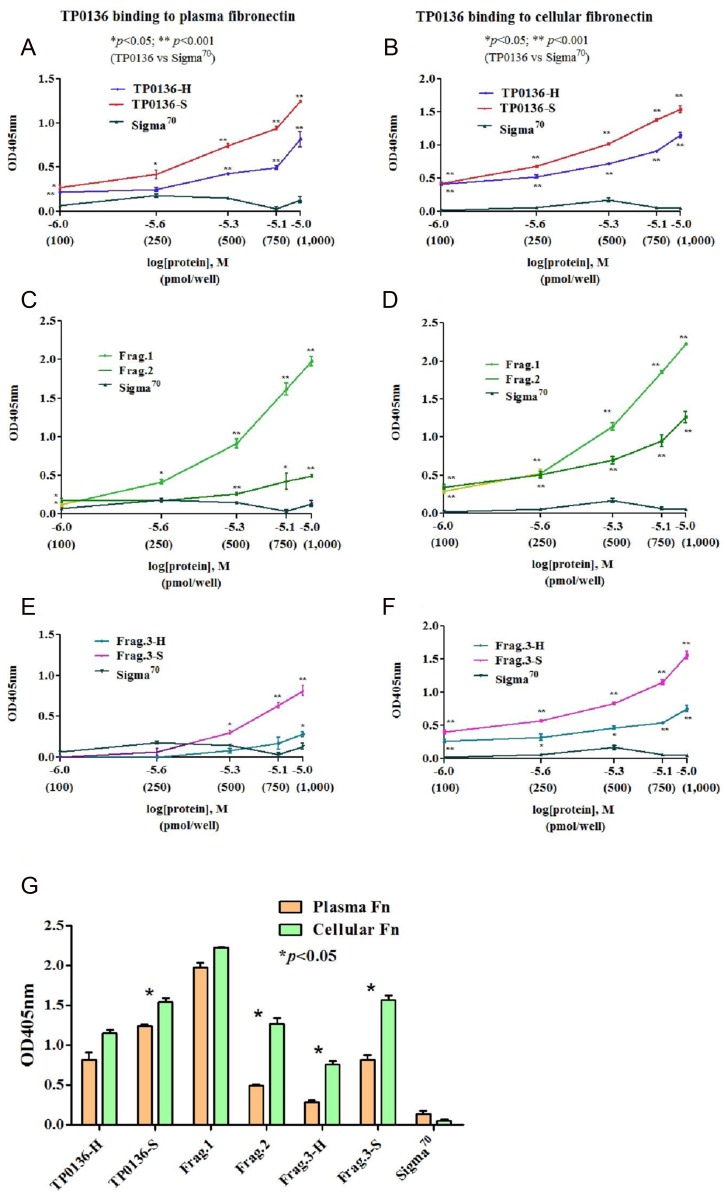
Recombinant TP0136 binding to plasma and cellular fibronectin. Adhesion to plasma and cellular Fn of TP0136 variants was evaluated using full-length recombinant TP0136 (w/o signal peptide) from Nichols Houston and Nichols Seattle, as well as four additional recombinant proteins representing the NH_2_-terminal (Frag.1), central (Frag.2), and COOH-terminal regions (Frag.3-H and Frag.3-S) of both TP0136 variants. *T*. *pallidum* transcription factor σ^70^ served as negative control. Each experiment was repeated three times to ensure reproducibility of results. Each time, each sample was tested in triplicate. (A-F) Results of the binding assay to plasma and cellular Fn. Colors represent different recombinant proteins used to assess dose-dependent binding to plasma Fn. X axis data point correspond to 100 pmol of protein/well (log[protein] = -6.0), 250 pmol/well (-5.6), 500 pmol/well (-5.3), 750 pmol/well (-5.1), and 1000 pmol/well (-5.0). In all panels, data points represent the absorbance at 405 nm ± SEM for triplicate samples. Significance was assessed by ANOVA for data in panel A-F (*p<0.05; **p<0.001) (G) Comparison between binding of recombinant proteins to plasma and cellular Fn. Data in Fig. 3G represent binding to Fn variants of 1,000 pmol of protein from panels above. Data points represent the absorbance at 405 nm ± SEM for triplicate samples. Significance was assessed by Student’s unpaired two-tailed t test with significance set at *p<*0.05 (*).

Recombinant full-length TP0136-H and TP0136-S variants, as well as Frag.1, Frag.2, Frag.3-H, and Frag.3-S protein fragments (red, orange, and green arrows in Fig. 1) from both variants were tested for their ability to bind plasma and cellular Fn. Results are shown in Fig. 3, panels A-F. Compared to the negative control σ^70^, both full-length variants significantly bound plasma and cellular Fn in a dose-dependent manner (Fig. 3A and B). For the individual fragments, Frag.1 and Frag.2 (conserved in both variants) demonstrated dose dependence with both plasma and cellular Fn, although Frag.1 exhibited significantly higher binding activity than Frag.2 (Fig. 3C and D). Frag.3-S also showed dose-dependent binding of both plasma and cellular Fn (Fig. 3E and F). Frag.3-H showed significant dose-dependence for binding to cellular Fn at all concentrations, but only at the highest concentration with plasma Fn. These data are reproduced in Fig. 3G to better demonstrate that, at the highest concentration tested, all recombinant proteins except TP0136-H and Frag.1 showed higher binding to cellular Fn than to plasma Fn. For both of these proteins, binding to both types of Fn was significantly higher than to the negative control.

### Recombinant TP0136 proteins inhibit attachment of *T*. *pallidum* to plasma and cellular fibronectin

**Fig 4:**
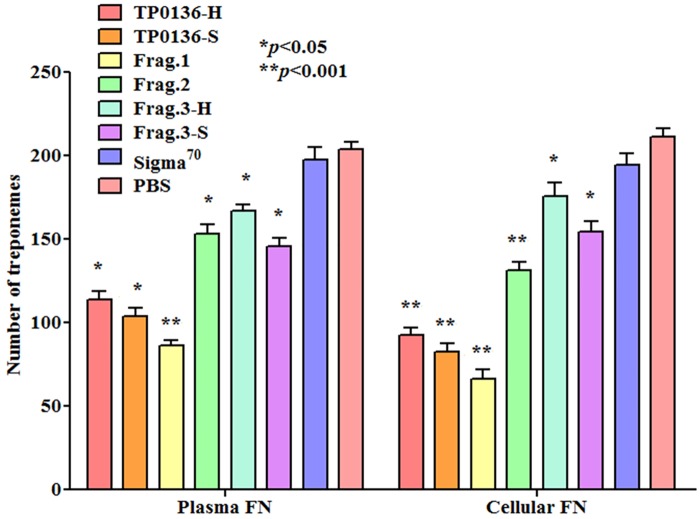
Inhibition of T. pallidum attachment to plasma and cellular fibronectin by recombinant TP0136 proteins. T. pallidum attachment to plasma and cellular fibronectin-coated slides is inhibited by slide pre-incubation with 800 pmol/well of recombinant proteins based on the TP0136 sequences form Nichols Houston and Nichols Seattle. T. pallidum σ70 and PBS were used as negative controls. Experiments were repeated twice using each time triplicate wells per condition. Bars represent the mean number of T. pallidum cells counted in 10 fields of triplicate experiments ± standard error. Significance was assessed by comparing wells coated with recombinant proteins to the PBS control wells with Student’s unpaired two-tailed t-test and significance set at p≤0.05. For comparison between different protein concentrations ANOVA test was used (*p<0.05; **p<0.001).

Because the recombinant proteins and peptides bind Fn directly, we tested whether these recombinants could block the attachment of *T*. *pallidum* to Fn. As shown in Fig. 4, pre-incubation of recombinant TP0136 proteins and peptides with Fn significantly inhibits the attachment of *T*. *pallidum* (Nichols Seattle) to both plasma and cellular Fn, compared to the PBS control. As expected, pre-incubation of Fn with the negative control recombinant σ^70^ protein did not significantly decrease treponemal attachment compared to PBS (Fig. 4). Among the individual fragments tested, Frag.1 was the most efficient in blocking treponemal adhesion to both plasma and cellular Fn (Fig. 4). Inhibition of binding of *T*. *pallidum* to plasma and cellular Fn was dose-dependent for all recombinant proteins except Frag.3-H variant of TP0136 (when tested with plasma Fn), or Frag.3-H and Frag.3-S when tested with cellular Fn (S1 Dataset).

### Anti-TP0136 rabbit immune sera demonstrate strain specificity and inhibit attachment of *T*. *pallidum* to Fn

**Fig 5:**
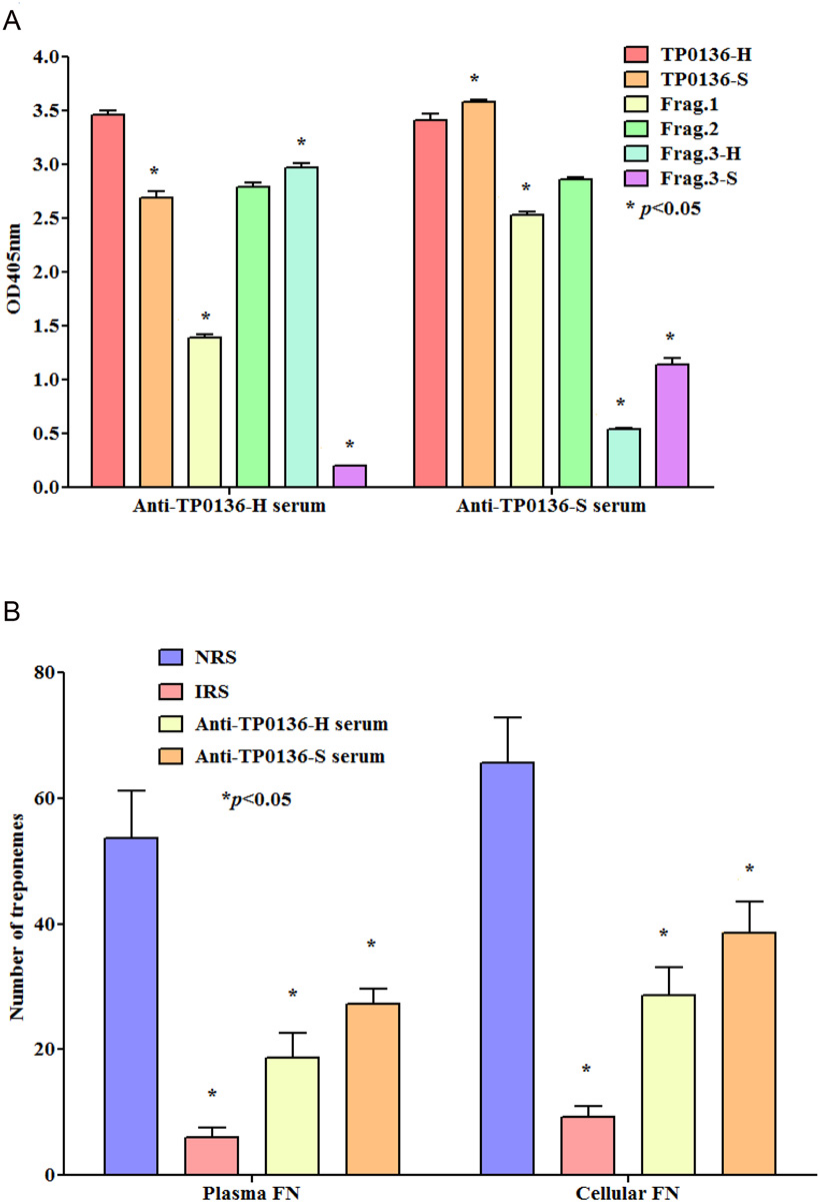
Reactivity of anti-TP0136 immune sera against recombinant TP0136 proteins and Inhibition of *T*. *pallidum* attachment to plasma and cellular fibronectin by anti-TP0136 immune sera. (A) Sera obtained from TP0136-H and TP0136-S-immunized rabbits recognized both recombinant full-length proteins and Frag. 1 and Frag. 2. Anti-TP0136-H antiserum recognized Frag.3-H but reacted only weakly against Frag.3-S. Similarly, anti-TP0136-S antiserum recognized Frag.3-S but reacted less well against Frag F3-H. Bars represent the absorbance at 405 nm ± SEM for triplicate samples. Significance between reactivity to single peptides was assessed by Student’s unpaired two-tailed t test with significance set at * *p<*0.05. (B) Antisera directed against TP0136-H and TP0136-S inhibited treponemal attachment to slides coated with plasma and cellular Fn. Experiments were repeated twice using each time triplicate wells per condition. Bars represent the mean number of *T*. *pallidum* cells counted in 10 fields of triplicate experiments (± standard error) following incubation of *T*. *pallidum* with either anti-TP0136 immune sera, IRS (positive control) or NRS (negative control). Significance is calculated with respect to NRS using the Student two-tailed t test with significance set at * *p<*0.05.

Following immunization with TP0136-H and TP0136-S, analysis of reactivity of rabbit antisera raised against recombinant full-length proteins showed that the antibodies raised against TP0136-H recognized all recombinant TP0136 proteins but, as expected, had very limited cross-reactivity to Frag.3-S. Similarly, anti TP0136-S reacted well to all homologous peptides, but had limited cross-reactivity against Frag.3-H (Fig. 5A).

We reasoned that, if TP0136 is expressed on the surface of viable *T*. *pallidum*, antibodies directed against Fn-binding regions of TP0136 should block the ability of *T*. *pallidum* to bind Fn in our *in vitro* assay. Compared to NRS, both immune sera against TP0136-H and TP0136-S showed a significant ability to inhibit treponemal attachment to plasma and cellular Fn-coated surfaces (Fig. 5B). As expected, IRS obtained from *T*. *pallidum*-infected rabbits was more effective than anti-TP0136 at inhibiting the binding of treponemes to Fn (*p* values ranged between 0.04 and 0.001), probably because IRS also contains antibodies to additional Fn-binding proteins (e.g. TP0155 and TP0483) expressed by *T*. *pallidum*.

## Discussion

Pathogen adhesion to host tissue components is a critical initial step of the process that leads to the establishment of infection. For this purpose, bacteria have evolved adhesins that specifically interact with host proteins (such as Fn, laminin, collagen, and elastin) that together with proteoglycans constitute the main ECM components. Fn is among the most common targets for bacterial adhesins [38,39]. The ability to bind Fn was reported for the syphilis agent over three decades ago by Peterson *et al*. [18] and by Fitzgerald *et al*. in presentations at the UCLA Symposium on the Molecular Biology of Host-Parasite Interaction and published shortly thereafter [19]. Peterson’s work showed that *T*. *pallidum* was capable of binding Fn and that pathogen adherence to Fn-coated coverslips or Fn-producing cell monolayers was significantly inhibited by anti-Fn antibodies [18]. Furthermore, based on the results of affinity chromatography of *T*. *pallidum* proteins using Fn as bait, it was concluded that at least three proteins (named P1–3) would mediate Fn binding *in vivo*. Although the genes for two of these Fn receptors were later cloned [40,41] and used to express recombinant proteins in *E*. *coli*, gene sequencing was not performed and, aside from their molecular size, their identity remained elusive. In their early work, Fitzgerald *et al*. [19] reported *T*. *pallidum* adherence to coverslips coated with Fn, laminin, collagen I and IV, and that treponemal attachment to such components could be inhibited by prior *T*. *pallidum* exposure to IRS. Collectively, these studies established for the first time the specific nature of the *T*. *pallidum-*Fn interaction. Subsequently, in an attempt to explore the specificity of *T*. *pallidum*-Fn interaction, Thomas *et al*. [42,43] showed that 1) attachment of *T*. *pallidum* to Fn-coated slides was inhibited by antibodies directed against the Fn cell- (or integrin-) binding-domain and, to a lesser extent, by antibodies targeting the heparin-binding domain, and that 2) the RGDS (arg-gly-asp-ser) sequence of the cell-binding domain was critical for *T*. *pallidum’s* binding to Fn. Finally, Fitzgerald and Repesh [44] reported that “young” treponemes (harvested from the rabbit host at day 7 post-inoculation) would adhere to Fn more efficiently than 14-day “old” treponemes, and hypothesized that *T*. *pallidum*’s ability to bind Fn could vary during the course of infection. Following these initial findings, the interest in *T*. *pallidum*’s adhesion to host ECM abated, to be subsequently re-ignited by the sequencing of the Nichols strain genome of *T*. *pallidum* [20] in the late 1990’s. This facilitated the identification of *T*. *pallidum* putative surface-exposed proteins using *in silico* approaches and led Cameron *et al*. to identify the TP0155 and TP0483 proteins as Fn receptors [22]. A similar role was reported for TP0136 by Brinkman *et al*. [21]. Overall, these findings confirmed the redundancy of proteins with Fn-binding activity in *T*. *pallidum*. Such redundancy is a trait common to many bacterial pathogens, and is the likely cause of variable results when virulence of wild-type and mutant strains for Fn-binding proteins are compared in pathogens that, unlike *T*. *pallidum* are amenable to genetic manipulation [45,46,47,48,49]. Similarly, as shown by our data, antibodies directed against the full-length TP0136 variants studied here did not abrogate *T*. *pallidum* ability to adhere to Fn as efficiently as IRS (Fig. 5B), suggesting that upon blocking TP0136 activity, binding to Fn still occurs via other adhesins.

At the molecular level, only the interaction between TP0483 and Fn was partially characterized by Dickerson *et al*. [50], who reported that two discrete regions of 16 (QMHSDS-KQVDVKLDGN) and 18 (QRKEDDSMYSYVTGTMKY) aa, respectively, located in the protein’s COOH-terminal region mediate binding to Fn, and that binding could involve one or both the cell- and gelatin-binding domains of Fn. To shed light on the TP0136-Fn interaction dynamics, we initially compared the TP0136 sequences from *T*. *pallidum* isolates, with the goals of 1) assessing the level of sequence conservation of TP0136 among strains and 2) identifying regions of sequence conservation to target for our study in case of marked variability. The choice to focus on the TP0136 variants from the Nichols Seattle and Nichols Houston lineages was made because, due to a nucleotide insertion in the Nichols Seattle gene, these variants completely differ from each other at their COOH-termini but share identical NH_2_-termini (Fig. 1).

Overall, our results suggest that the conserved region encompassing aa 32–234 (Fragment 1) is primarily responsible for binding to plasma Fn. TP0136’s major binding activity to plasma Fn should be exerted by one or more sequences found within the first 201 aa of the mature peptide (w/o the putative cleavable signal peptide). Two identical repeats in the TP0136 NH_2_-terminal region (highlighted by black double headed arrows in Fig. 1) are reminiscent of the repeats that mediate binding to the Fn type I module of Fn in staphylococcal and streptococcal adhesins [51]. Although limited sequence homology between those and the TP0136 repeats, binding to Fn might not necessarily involve a specific sequence on the receptor, but rather the presence of appropriate secondary structures or amino acid regions able to transition from an intrinsically disordered structure to a defined one that can successfully bind Fn. Such a theory is supported by structural studies conducted using the Fn type I module and the B repeat of the *Streptococcus dysgalactiae* (*S*. *dysgalactiae*) Fn-binding protein B (FnBB) [52]. The Fn type I module fold consists of a β-hairpin stacked onto a triple-stranded antiparallel β-sheet. When the B repeat of FnBB interacts with two Fn type I modules, the sequence of the B repeat transitions from a disordered structure to an ordered one, to form a fourth antiparallel strand that interacts with the triple-stranded β-sheet of Fn type I modules. This interaction forms a so called “tandem β-zipper” structure that is responsible for binding to Fn [52]. A similar interaction between TP0136 repeats and Fn remains speculative and requires additional work to be confirmed, given that no binding experiments were performed in this study using single Fn modules or peptides representing only the TP0136 repeats.

A different hypothesis to explain the high affinity of Fragment 1 for plasma Fn comes from the evidence that a short 16 aa sequence in the TP0136 NH_2_-terminus (PTDSDKKVMSIATDGN, upstream of the repeats) shares 37.5 identity and 43.7 similarity with the TP0483 sequence reported by Dickerson *et al*. to interact with the Fn cell-binding domain [50]. This could suggest that Fn-TP0136 interaction dynamics is not dissimilar from that of TP0483. Further studies will determine whether TP0136 and TP0483 target different regions of the Fn molecule or, instead, compete for the same target.

Surprisingly, the COOH-terminal region from Nichols Seattle showed binding activity to plasma Fn while the corresponding region from Nichols Houston was less effective. This result is intriguing given that the Nichols Seattle fragment harbors sequences normally found within the variable regions of TprK [37], a surface protein believed to be involved in *T*. *pallidum* immune evasion and persistence in the host [53]. TprK undergoes antigenic variation in seven discrete variable (V) regions through a non-reciprocal gene conversion mechanism that exploits donor sequences located approximately 314 Kbp from the *tprK* expression site [54]. According to current 3D models, TprK is a β-barrel outer membrane protein with the seven V regions located in the protein surface-exposed loops, consistent with a role for these changing sequences in immune evasion [53]. Although TprK’s ability to adhere to ECM components is yet to be addressed experimentally, this protein shares sequence homology with the major sheath protein (Msp) of the oral pathogen *T*. *denticola*, known to be a virulence factor able to bind Fn, fibrinogen, and laminin [55] but also keratin, type I collagen, heparin and hyaluronic acid [56]. Our data therefore could be preliminary evidence that TprK is also involved in binding to the host ECM. According to 3D models for TprK, no defined secondary structure is associated with the V region loops. However, in the last decade reports have increased of proteins with disordered regions that transition to an ordered structure upon binding to a target. These studies included Fn-binding proteins of *S*. *aureus* and *S*. *dysgalactiae* as well as Fn itself, reported to adopt a more extended conformation upon interaction with Fn binding proteins [57,58,59,60].

It is more complex to interpret the results of the binding assays of full-length TP0136 variants and protein fragments to cellular Fn, which also showed significant binding by Fragments 2 and 3 from Nichols Houston (Fig. 3D and 3F), in spite of their weak or absent binding, respectively, for plasma Fn. A possible explanation might be found in the differences between plasma and cellular Fn. Fn is composed of repeated modules: type I modules (12), type II modules (2), type III modules (17), and a type III connecting segment. In plasma Fn, produced by the liver, only one subunit carries a type III connecting segment, and two type III modules are absent [61,62,63]. Cellular Fn is synthesized by several cell types, including fibroblasts, endothelial cells, chondrocytes, synovial cells, and myocytes [64] and is a mixture of different Fn isoforms that derive from the alternative splicing of two type III modules and of the type III connecting segment [65,66,67]. Because the flexibility of Fn type III modules is affected by neighboring domains, alternatively spliced domains may change the global conformation of Fn and perhaps expose additional target sequences for TP0136.

Our data seem in contrast with the early observation that 14-day “old” *T*. *pallidum* cells had reduced binding capacity to Fn compared to “young” treponemes harvested seven days post-inoculation [44]. Our results, in fact, suggest that TP0136 transcription is steady at these two time points, increasing later during experimental infection. A decrease in Fn binding ability between these two treponemal populations [44] could be due to post-transcriptional regulatory mechanisms that affect negatively protein synthesis, in spite of the presence of message. An additional explanation, however, could be found in the possibility that the longer the treponemes grow in the rabbit host the more their surface becomes coated with free ECM components and antibodies against surface antigens. This phenomenon, in turn, could account for a diminished phenotypic ability to recognize and bind to ECM *in vitro* by “older” *T*. *pallidum*. The increase in TP0136 transcription later during infection, on the other hand, could be important to allow the pathogen to bind plasma Fn during later stages, and to protect this surface antigen from antibody binding.

Finally, it is still unclear whether the putative P1–3 Fn-binding proteins identified by Peterson *et al*. in the early 1980’s [40,41] correspond to the *T*. *pallidum* proteins known today to bind Fn. Their proteins had molecular weights (MW) of 89.3, 37.0, and 32.0 kDa, respectively. The three known Fn-binding proteins in *T*. *pallidum* (TP0136, TP0155, and TP483) have MW of 50.1, 40.6, and 41.8 kDa, respectively. This could suggest that additional *T*. *pallidum* Fn-binding proteins wait to be discovered.

## Conclusions

The major conclusions of our study are that 1) TP0136 proteins are heterogeneous in sequence among *T*. *pallidum* isolates; 2) both Nichols Seattle and Nichols Houston TP0136 variants bind human cellular and plasma Fn, but recognize cellular Fn more efficiently than plasma Fn; 3) TP0136 NH_2_-terminus (Frag.1, in this study, identical in both variants analyzed) exerts the major Fn binding activity, even though binding regions for cellular Fn appear to be scattered throughout the entire protein length, and 4) *tp0136* transcription is ongoing during experimental infection with increased levels seen during the healing of the primary chancre. Altogether, these data support the importance of TP0136 role in syphilis pathogenesis.

## Supporting Information

S1 Dataset Inhibition of binding of T. pallidum to plasma and cellular Fn. Inhibition of binding of T. pallidum to plasma and cellular Fn is dose-dependent for all recombinant proteins except Frag.3-H variant of TP0136 (when tested with plasma Fn, Panel A), or Frag.3-H and Frag.3-S when tested with cellular Fn (Panel B). In both experiment 0.5 μg of Fn were added to each experimental well. * p<0.05; ** p<0.001
(PDF)
