# Supplementary material for: Treponema pallidum subsp. pallidum TP0136 Protein Is Heterogeneous among Isolates and Binds Cellular and Plasma Fibronectin via its NH2-Terminal End
Source: PLoS Negl Trop Dis. 2015 Mar 20;9(3):e0003662. doi: 10.1371/journal.pntd.0003662 (PMC4368718; doi:10.1371/journal.pntd.0003662)
Supplement: S1 Dataset — Inhibition of binding of T. pallidum to plasma and cellular Fn is dose-dependent for all recombinant proteins except Frag.3-H variant of TP0136 (when tested with plasma Fn, Panel A), or Frag.3-H and Frag.3-S when tested with cellular Fn (Panel B). In both experiment 0.5 μg of Fn were added to each experimental well. * p<0.05; ** p<0.001 (PDF) [file pntd.0003662.s001.pdf]

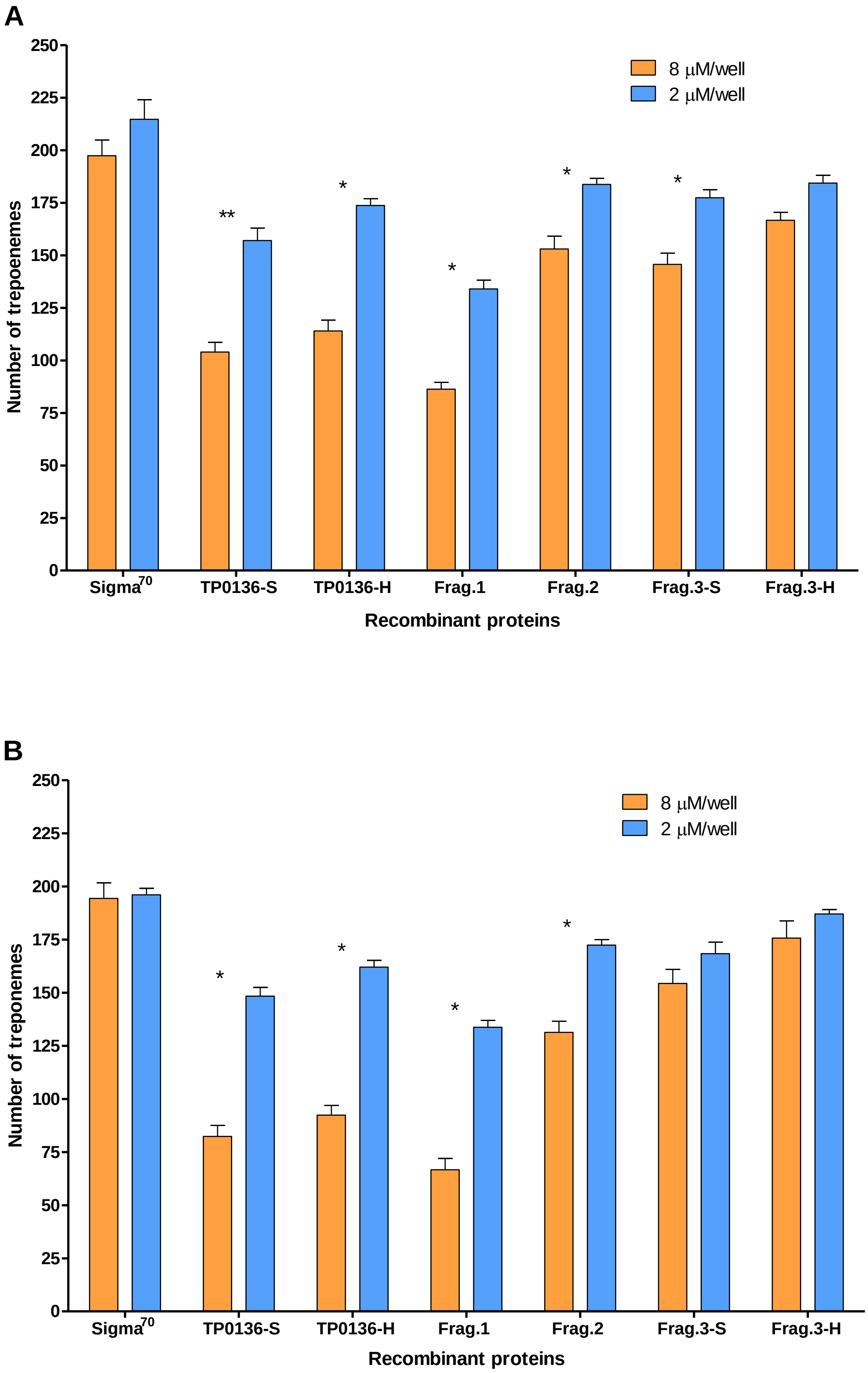
A
8 μM/well
2 μM/well
Number of trepoenemes
Recombinant proteins
Sigma70
TP0136-S
TP0136-H
Frag.1
Frag.2
Frag.3-S
Frag.3-H
B
8 μM/well
2 μM/well
Number of treponemes
Recombinant proteins

Inhibition of binding of *T. pallidum* to plasma and cellular Fn is dose-dependent for all recombinant proteins except Frag.3-H variant of TP0136 (when tested with plasma Fn, Panel A), or Frag.3-H and Frag.3-S when tested with cellular Fn (Panel B). In both experiment 0.5 μg of Fn were added to each experimental well. * $p<0.05$; ** $p<0.001$
